# Photobiomodulation Therapy in the Management of Oral Lichen Planus: A Systematic Review and Meta-Analysis

**DOI:** 10.1055/s-0044-1782213

**Published:** 2024-05-14

**Authors:** Wei Kang Soh, Kwok Fu Cheah, Sajesh K. Veettil, Deepak Pandiar, Smita Nimbalkar, Divya Gopinath

**Affiliations:** 1 School of Dentistry, International Medical University, Kuala Lumpur, Malaysia; 2 Department of Pharmacy Practice, School of Pharmacy, International Medical University, Kuala Lumpur, Malaysia; 3 Saveetha Dental College and Hospitals, Saveetha Institute of Medical and Technical Sciences, Saveetha University, Chennai, India; 4 Clinical Oral Health Sciences, International Medical University, Kuala Lumpur, Malaysia; 5 Basic Medical and Dental Sciences Dept, College of Dentistry, Ajman University, United Arab Emirates; 6 Centre of Medical and Bio-allied Health Sciences Research, Ajman University, Ajman, United Arab Emirates

**Keywords:** photobiomodulation therapy, oral potentially malignant disorder, lichen planus, OLP, low-level laser therapy

## Abstract

Photobiomodulation therapy (PBMT) is a non-invasive and the latest form of therapy used in the treatment of non oncological diseases as well as cancers of various types and locations. The aim of this study was to systematically review and assess the efficacy of PBMT in managing oral lichen planus (OLP) compared to the interventions. A systematic review and meta-analysis were implemented according to the Preferred Reporting Items for Systematic Reviews and Meta-Analyses (PRISMA) guidelines. An electronic search using PubMed, Scopus, and Cochrane was conducted to retrieve relevant studies published until June 2023. The outcomes evaluated included the reduction in pain score and clinical severity scores (Prospero No CRD42023428626). A total of eight studies were identified for qualitative synthesis. The pooled analysis incorporating six studies revealed that there are no significant differences for both mean pain score (mean difference [MD] = 0.21, 95% confidence interval [CI] = −0.51, 0.93) as well as clinical score (MD = −0.08, 95% CI = −0.4, 0.25) between PBMT and comparison groups. Subgroup analysis based on corticosteroids as controls showed that there was no significant difference in mean reduction in pain score between PBMT and topical steroids (MD = 0.38, 95% CI = −0.54, 1.31). PBMT is as effective as other interventions in the treatment of OLP, though not superior, and can be a promising alternative treatment for cases resistant to steroids or when steroids are contraindicated. Further studies are recommended to standardize the optimal settings for the treatment of OLP.

## Introduction

Photobiomodulation therapy (PBMT) is widely used in the treatment of various diseases, including ophthalmology-related diseases, vascular-endothelial-cells-related diseases, acne, and even cancers. 1 PBMT, previously known as low-level laser therapy, utilizes laser or non-ionizing radiation, including light-emitting diodes, in the visible (400–700 nm) and near-infrared (700–1100 nm) electromagnetic spectrum. During PBM therapy, photons penetrate the tissue and interact with the mitochondrial cytochrome c complex, which sets off a series of biological processes that improve cellular metabolism, which can both lessen pain and hasten the healing process. 2

Oral lichen planus (OLP) is a chronic immune-mediated, inflammatory, and psychological illness that usually affects the oral mucosa in a characteristic bilateral pattern. 3 The prevalence of OLP worldwide is 2.2%. Patients with erosive-atrophic variants of OLP, which appear as diffuse, erythematous patches encircled by thin white lines (Wickham striae), frequently seek therapy since these lesions are painful and uncomfortable. 4 5 Wickham striae are the white striations seen essentially in reticular OLP and can be, but not always, found surrounding erosive OLP. Some lesions may develop into malignant transformations in erosive atrophic patterns, hence classified as an oral potentially malignant disorder. 6 Even though there are widely accepted, conservative/pharmacological therapeutics available for OLP, they are time-consuming, and recurrences of these lesions are common even after the therapy is ceased. The search for better and advanced treatment alternatives has led to the emergence of new treatment approaches for these lesions, including various forms of phototherapy.

Several trials have concluded that PBMT can produce notable relief in the signs and symptoms and an increase in the symptom-free periods in OLP and thus can be considered an effective and safe advanced treatment modality for OLP. 7 8 9 It is minimally invasive as it has selective toxicity toward target tissues and provides good cosmetic results with little or no scarring. 10 11 In the available literature, two sessions of PBMT per week show promising results in severe symptoms. Still, more often, three appointments per week or daily PBMT for the first 5 days and then every other day is recommended. 12 A review published in 2017 by Al-Maweri et al emphasized that PBMT is effective in the management of symptomatic OLP. However, another systematic review by Akram et al in 2018 that sought to assess the efficacy of PBMT in comparison to topical corticosteroids in the therapy of atrophic-erosive types of OLP concluded that it remains debatable whether PBMT is more effective than topical corticosteroids. 13 Thus, the literature regarding the efficacy of PBMT in the management of various OLP is still inconclusive. 14 Moreover, there are no proper recommendations for the practitioner to follow in managing OLP. The present meta-analysis aims to systematically summarize the current evidence on the effectiveness of PBMT in the treatment of patients with OLP. This would also help the practitioners decide on the type of nonsurgical intervention to use when managing OLP, especially in the long term.

## Methods

### Registration

The protocol of systematic review was registered in the International Prospective Register of Systematic Review (PROSPERO NO CRD42023428626).

### Study Design

A systematic review and meta-analysis of the efficacy of PBMT on the management of OLP was implemented according to the general principles of the Cochrane Handbook for Systematic Reviews of Interventions and was reported according to the Preferred Reporting Items for Systematic Reviews and Meta-Analyses (PRISMA). 15 16

### Search Strategy

The relevant studies were identified through a systematic search of PubMed, Scopus, and Cochrane. A search for human studies published until June 2023 in the English language was performed by using two sets of search terms, one for the interventions including “low-level laser therapy,” “laser phototherapy,” “photobiomodulation therapy,” “laser therapy,” “laser treatment,” “diode laser” and the term used to describe the condition that included “oral potentially malignant disorder,” “oral precancer,” “oral premalignant,” “lichen planus,” “leukoplakia.” The Boolean operators OR and AND were used to combine these terms accordingly. We developed the search strategy for PubMed and modified it for other databases. The detailed search strategy is provided as Supplementary Tables S1–S3 (available in the online version). After removing duplicates, the titles and abstracts were screened against the predetermined eligibility criteria to decide whether to include them for further full-text reading. The record was subjected to full-text reading if the abstract provided a clear explanation regarding inclusion or exclusion. In addition, manual searches of relevant reviews reference lists were conducted to exclude the possibility of omitting any critical study.

### Study Selection

#### Inclusion Criteria

Randomized controlled trials (RCTs) or observational studies that meet the following inclusion criteria were included:

Population: Adults with OLP;
Intervention: PBMT for the management of OLP;
Comparison: Any other treatments;
Outcomes: The primary outcome was the resolution of pain measured in terms of the visual analog scale (VAS). The change in clinical severity score was selected as the secondary outcome.

#### Exclusion Criteria

Non-English literature, case reports, abstracts, and conference reports were excluded.

### Data Extraction and Quality Assessment

Titles and abstracts were screened independently by two reviewers (SWK and CKF) to evaluate the eligibility of all the retrieved studies, followed by full-text reading. To improve the sensitivity, papers were excluded if both authors eliminated them based on the title and abstract, and disagreements were resolved by discussion with a third author (DG). Data were extracted independently and duplicated into a data collection form by two reviewers. The extracted data was entered into the data collection form according to the following sections: Study characteristics, population characteristics, intervention characteristics, and outcome definitions and measures. For risk of bias assessment, two reviewers evaluated RCTs independently using the Cochrane risk of bias tool (ROB 2.0). 17 The Newcastle–Ottawa scale was used to assess the quality of observational studies. 18

### Data Synthesis

Meta-analysis was accomplished with DerSimonian and Laird random-effects model. Mean difference (MD) and 95% confidence intervals were utilized as outcome measures for both outcomes. The analysis was performed using the Stata version 15.0 (StataCorp, College Station, Texas, United States). 17 19 Heterogeneity between trials was assessed by considering the I ^2^ statistics; an I ^2^ estimate more than or equal to 50% was interpreted as evidence of substantial levels of heterogeneity. 17 Publication bias was assessed using a funnel plot. 20 Subgroup analyses were carried out based on the intervention characteristics. Sensitivity analysis was conducted exclusively on RCTs after the exclusion of the observational studies.

## Results

### Study Selection

**Fig. 1:**
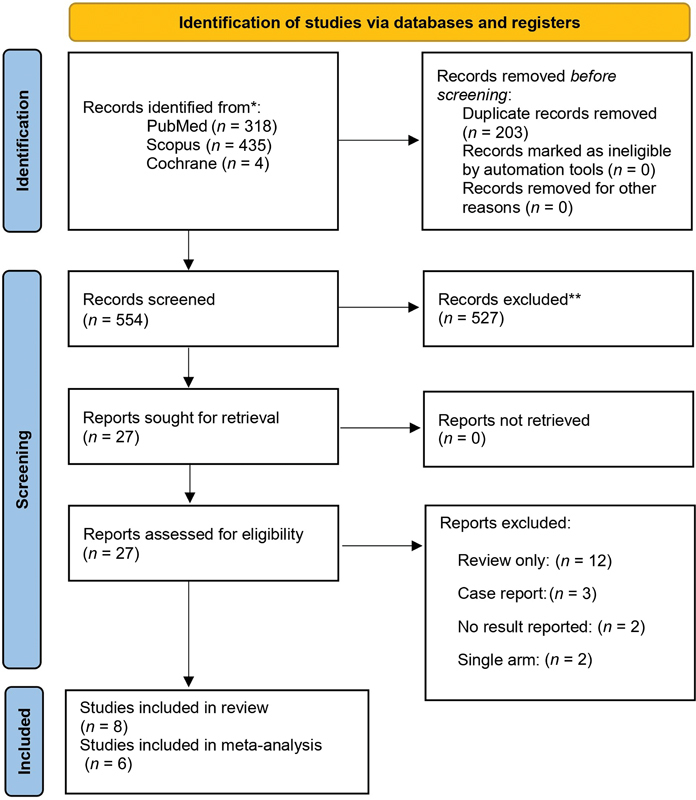
Preferred Reporting Items for Systematic Reviews and Meta-Analyses (PRISMA) flowchart illustrating the study screening and selecting process.

The detailed flow of the selection of studies for PBMT (PRISMA flowchart) is shown in Fig. 1 . The electronic searches of selected databases initially identified 757 studies. After removing 203 duplicates, 554 studies were obtained. Five hundred twenty-seven studies were further excluded after the title and abstract screening, yielding 27 articles. These 27 articles were assessed by full-text reading. The remaining 19 studies were excluded because 12 were reviews only, three were case reports, two had no reported results, and two were single-arm studies. Finally, only eight studies were included in qualitative synthesis as they reported the outcomes that fit our outcome criteria.

### Characteristics of the Included Studies

A total of eight studies were included for the qualitative synthesis with an enrolment of 317 patients with OLP. 21 22 23 24 25 26 27 28 Among these, seven studies were RCTs, while one was a case–control observational study. The studies were published in the English language between 2011 and 2022. Two of the studies were from India, 26 27 one from Egypt, 23 two from Brazil, 21 28 one from Iran, 25 one from Saudi Arabia, 22 and one from Turkey. 24 The number of randomized participants in the included studies ranged from 24 to 120. The age of the participants ranged from 18 to 63 years. The mean age of participants was 52.04 ± 6.55. Among the included studies, four studies used diode laser, 22 23 25 26 three studies used gallium-aluminum-arsenide laser (GaAlAs) 24 27 28 and one studies used aluminum-gallium- indium-phosphide laser (InGaAlP). 21 The control interventions tested were corticosteroids in five trials and photodynamic therapy, ozone therapy, and aloe vera each in single trial. The detailed characteristics are provided in Table 1 .

**Table 1:** Characteristics of the studies included in qualitative analysis

Reference	Region	Study design	Patient no /lesion no	Confounder	Lesion type and location	Diagnosis of lesion	Gender	Age, (years)	Intervention	Comparison	Follow-up period
Dillenburg et al (2014) 21	Brazil	RCT	42 patients (21 in each group)	None	Symptomatic atrophic/erosive OLP, tongue, buccal and labial mucosa, floor of mouth, gingiva, palate	Histopathology	7 males, 35 females	Clobetasol: 61.33 ± 11.85, PBMT: 55.14 ± 15.96	PBMT was administered using a continuous wave, InGaAlP diode laser diode laser with a wavelength of 660 nm, power output of 40 mW, output density of 1,000 mW∕cm ^2^ , energy density of 6 J∕cm2, 6-s exposure time per point, and 0.24 J of total. PBMT was administered 3x/week for 4 weeks, totaling 12 sessions.	Clobetasol propionate gel 0.05%, 3x/day applications for 30 days, candidiasis prevention was done with nystatin application	Follow-up weekly until a month and 4 weeks and 8 weeks after completion of treatment
Mirza et al (2018) 22	Saudi Arabia	RCT	45 patients (divided into 3 equal groups)	None	Erosive-atrophic OLP; tongue, buccal mucosa	Histopathology	8 males, 37 females	Group 1 (52.6 ± 11.4), Group 2 (50.8 ± 14.7), Group 3 (49.2 ± 10.6)	• Group-2 (low level laser therapy): diode laser, 1.5 J/cm2 per session; 2 times/week for max 10 sessions	• Group-1 (toluidine blue-PDT): topical 1mg/ml toluidine blue followed by GaAlAs laser (630 nm, 1.5 J/cm2 per session); 2 sessions, 2x/week for 1 month • Group-3 (control group): Topical dexamethasone	Follow-up weekly until a month and 1 year after completion of treatment
El-Shenawy and Eldin (2015) 23	Egypt	NRCT	24 patients (12 in each group)	2 patients are hypertensive, 1 patient is diabetic, and 4 patients are diabetic and hypertensive	Erosive-atrophic OLP, site not mentioned	Histopathology	5 males, 19 females	PBMT: 53.6 ± 13.2, CORT: 52.2 ± 6.4	12 patients were subjected to laser sessions with 970 nm diode laser, continuous non-contact mode with (320 µm) diameter fiber optic, 2x/week for max 10 sessions	12 patients treated with topical corticosteroids (0.1% triamcinolone acetonide orabase)	Follow-up weekly up to 4 weeks after completion of treatment
Kazancioglu and Erisen (2015) 24	Turkey	RCT	120 patients (divided into 4 equal groups)	None	Atrophic-erosive OLP, tongue or buccal mucosa	Clinically and histopathology	56 males, 64 females	42.6 ± 8.3	Group-1 (PBMT): GaAlAs laser (808 nm, 0.1 W, continuous wave) was used as a light source. A light exposure dose of 120 J/cm2 was used for 2.5 minutes, 2x/week for max 10 sessions	• Group-2 (ozone therapy): performed by using an ozone generator with a tissue probe, applied intraorally for 10s, 2x/week for max 10 sessions. • Group-3 (positive control): dexamethasone mouthwash for 5 minutes, followed 30 minutes later by 30 drops of nystatin solution, 4x/day for 1 month • Group-4 (negative control): A special solution filled with base ointment without the corticosteroid component was prepared, gargled solution for 5 minutes, 4x/day for 1 month	Follow-up at 1, 3, and 6 months after the treatment
Jajarm et al (2011) 25	Iran	RCT	24 patients for analysis, 30 recruited, randomly allocated		Atrophic-erosive biopsy-proven OLP in the tongue or buccal mucosa	Histopathology	Not mentioned	Not younger than 20	Group 1- A diode laser was used as a light source (Mustang2000 þ, Russia, KLO3 probe, 630 nm, 10 mW, continuous wave, spot size: 11 cm). Irradiation was done 2x/week for a maximum of 10 sessions	Group 2- dexamethasone (0.5 mg in 5 ml water) mouth wash for 5 minutes, followed 30 minutes later by a mouth rinse with 30 drops of Nystatin (100,000 units) for 5 minutes. This treatment was repeated 4x/day for 1 month	Follow-up weekly up to 1 month after completion of treatment
Jain et al (2021) 26	India	RCT	30 patients, 15 in each group	None	Symptomatic OLP, site not mentioned	Clinically and histopathology	10 males, 20 females	18-30	Group-1 (Steroid + PBMT): topical 0.1% triamcinolone acetonide oral base, 5 x/day for 28 days or till the lesions heal + PBMT delivered by the photon (3W) zolar diode laser with wavelength: 810 nm, mode: continuous defocused non-contact mode, power output: 0.8–0.9 W, time duration: 10 minutes laser equipment, 2x/week for 9 sessions	Group-2 (Steroid): topical 0.1% triamcinolone acetonide oral base, 5x/day for 28 days or till the lesions heal	Follow-up once every 15 days for 2 months after completion of treatment
Bhatt et al (2022) 27	India	RCT	60 patients (divided equally in to 2 groups)	None	Oral lichen planus	Clinically and histopathology	38 females, 22 males	Mean age of 40.73	GaAlAs diode laser (980 nm wavelength twice weekly for 2 months. 0.6 W/cm2, 12 J/cm2, twice weekly for 2 months	Aloe vera extract 500 mg capsule was mixed with carboxymethylcellulose powder, applied topically for 30 minutes, for 2 months	Follow-up weekly for 9 months after completion of treatment
Ferri et al (2021) 28	Brazil	RCT	34 patients, 17 in each group	None	Reticular-atrophic-erosive OLP, buccal mucosa, gingiva, tongue, palate, lips, alveolar ridge, floor of mouth	Histopathology	32 females, 2 males	mean age of 62.2	GaAIAs diode laser, with 660nm wavelength, irradiance: 35.4mW/cm2, radiant exposure: 177J/cm2, 5 sec exposure time per point and 0.5J of total energy per point, 2x/week for 4 weeks, for 8 sessions	Clobetasol propionate gel 0.05%, covering the OLP lesions completely, applied 3 x/day for 30 consecutive days	Follow-up at 60 days, 90 days, and 120 days after the treatment

Abbreviations: GaAIAs, gallium-aluminum-Arsenide laser; InGaAlP, aluminum-gallium- indium-phosphide laser; NRCT, nonrandomized controlled trial; OLP, oral lichen planus; PDT, Photodynamic therapy; PBMT, photobiomodulation therapy; RCT, randomized controlled trial.

### Risk of Bias Analysis

Seven of the included articles were RCTs. 21 22 24 25 26 27 28 The risk of bias was evaluated with the Cochrane risk-of-bias tool for randomized trials. 17 All studies had carried out proper sequence generation. Thus, the risk of bias that might arise from this domain was assessed as low. Only one study did not report the methods utilized to conceal the allocation process; therefore, the risk of bias was assessed as unclear for this domain. 25 Blinding of participants and personnel was not performed in the two studies. 21 22 Thus, the risk of bias is high in these studies. One of the studies 24 was categorized as an unclear risk of bias due to insufficient information provided by the authors to permit judgment. Blinding of the outcome assessment also did not occur in three of the studies due to the subjective elements involved in the outcomes. 21 27 28 The risk of attrition bias was low in most of the included studies, and only two had a high risk of attrition bias. 25 28 Five studies also had unclear chances of selective reporting. 21 22 24 26 28 The quality of three studies was poor, and only four were fair. One study was observational, and thus, the risk of bias was evaluated using Newcastle Ottawa Scale. 23 The study was classified as poor quality. The risk of bias is shown as Supplementary Tables S4 and S5 (available in the online version).

### Efficacy of PBMT on Pain Score (VAS)

**Fig. 2:**
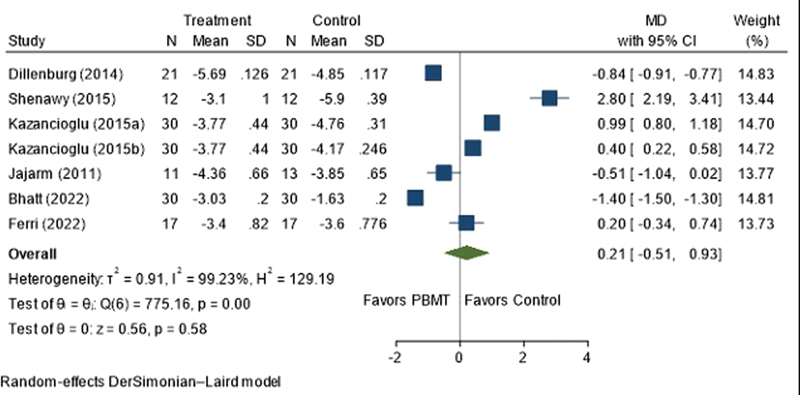
Forest plot illustrating pooled data on the efficacy of photobiomodulation therapy (PBMT) on pain score (visual analog scale). CI, confidence interval; MD, mean difference; PBMT, photobiomodulation therapy; SD, standard deviation.

**Fig. 3:**
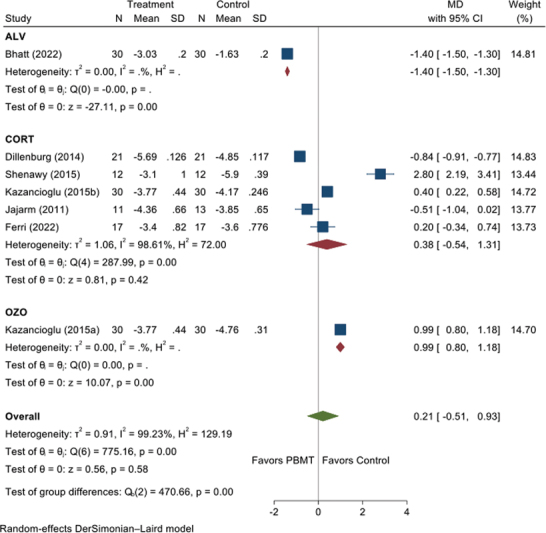
Forest plot illustrating sub-group analysis for pain score (visual analog scale). CI, confidence interval; MD, mean difference; PBMT, photobiomodulation therapy; SD, standard deviation.

**Fig. 4:**
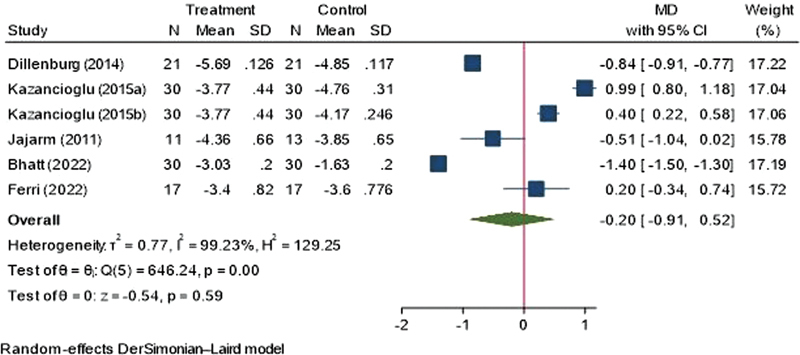
Forest plot showing sensitivity analysis for pain score (visual analog scale). CI, confidence interval; MD, mean difference; SD, standard deviation.

We extracted the data from six articles to assess the pain score. 21 23 24 25 27 28 The pooled meta-analysis showed that there was no statistically significant difference between the PBMT and control groups, with a MD of 0.21 (95% CI = −0.51, 0.93; Fig. 2 ). High heterogeneity was found between studies, with an I ^2^ of 99.23%, indicating a wide variation. The Funnel plot illustrated publication bias ( Supplementary Fig. S1 , available in the online version). The contour-enhanced funnel plot suggested missing studies on the regions of nonsignificance, showing publication bias. ( Supplementary Fig. S2 , available in the online version). To explore the heterogeneity, we performed subgroup analyses based on the control group ( Fig. 3 ). There were five studies on the comparison between topical corticosteroids. However, there was no significant difference in mean reduction in pain score between PBMT and topical steroids (MD = 0.38, 95% CI = −0.54, 1.31). Sensitivity analysis was performed on studies that are exclusively RCTS. There was no statistically significant difference in the mean reduction in pain between the PBMT groups and control groups (MD = 0.20, 95% CI = −0.91, 0.52; Fig. 4 ).

### Efficacy of PBMT on a Clinical Severity Score

**Fig. 5:**
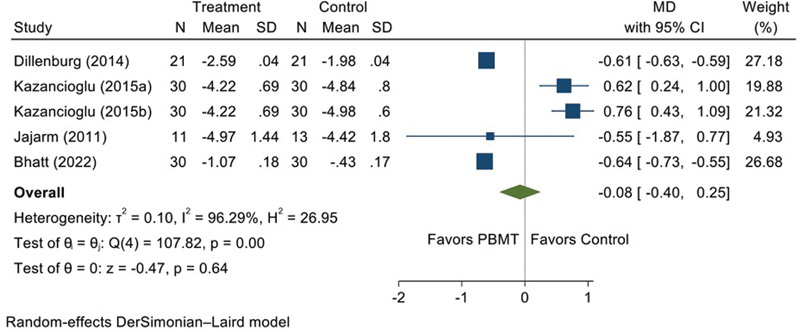
Forest plot illustrating pooled data on the efficacy of photobiomodulation therapy (PBMT) on clinical severity score. CI, confidence interval; MD, mean difference; PBMT, photobiomodulation therapy; SD, standard deviation.

**Fig. 6:**
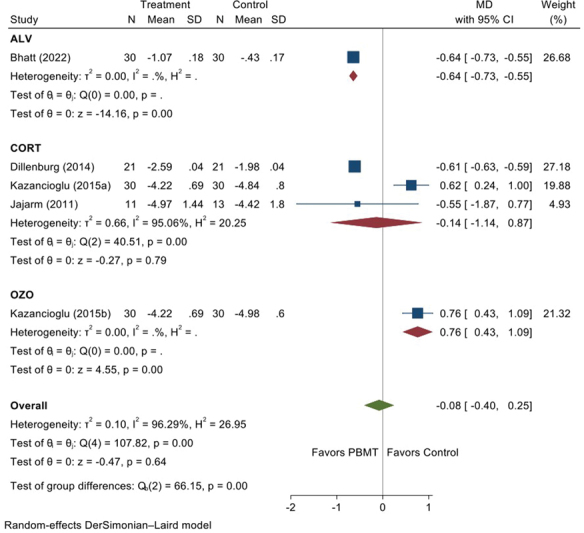
Forest plot illustrating sub-group analysis for clinical severity score. CI, confidence interval; MD, mean difference; PBMT, photobiomodulation therapy; SD, standard deviation.

Only four studies (RCTs) were found to assess clinical severity. 21 24 25 27 No statistically significant differences were identified between the PBMT and control treatment employed (MD = −0.08, 95% CI = 0.4, 0.25; Fig. 5 ). High heterogeneity was found between studies, with an I ^2^ of 96.29%, indicating a wide variation. Additionally, the funnel plot asymmetry test publication bias. The funnel plot and contour-enhanced funnel plot are provided as Supplementary Figs. S3 and S4 (available in the online version), respectively. To explore the heterogeneity, we performed subgroup analyses based on the control group. There were three studies on the comparison between topical corticosteroids and PBMT. However, there was no significant difference in mean reduction in pain score between PBMT and topical steroids (MD = −0.14; 95% CI = 0.40, 0.25; Fig. 6 ).

## Discussion

In recent years, developments of lasers in dentistry have encouraged the use of PBMT as a practical treatment option for several oral diseases. In this study, we focused on assessing the effectiveness of PBMT in the management of OLP. Our results highlight that PBMT is as effective as any other control treatment, including corticosteroids.

A previous meta-analysis that exclusively focused on the effectiveness of PBMT in comparison with corticosteroids also concluded that it is a reliable alternative to corticosteroids. However, in contrast, our study has included all the tested interventions that have been compared with PBMT and also recently published additional studies in our meta-analysis. Another systematic review by Al-Maweri et al also highlighted the utility of PBMT in OLP; however, it did not perform quantitative analysis. 29 Thus, our study is the most updated and comprehensive evidence on the effectiveness of PBMT.

Dillenburg et al have reported that PBMT is a more effective intervention for the treatment of OLP. 21 These findings are also supported by Jain et al, and Bhatt et al. 26 27 On the other hand, five other trials have reported that PBMT is less effective than control interventions in improving pain scores. 22 23 24 25 28 Hence, the pooled analysis could not specify the superiority of one treatment over the other. The variation in individual trial results might be attributed to different parameters that affect the treatment, including wavelength, power, energy density, treatment duration and intervention time, method of application, structure, and condition of the tissue. 24 Most of those protocols included 8 to 12 PBM sessions to show comparable improvements in clinical symptoms. However, a recent paper reported that a single session of laser PBM may be safe and effective in reducing pain for symptomatic OLP patients. 30 However, the study did not have any control arm; hence, further, randomized controlled studies with placebo or topical corticosteroids as a comparison would be necessary to provide sound evidence on the utility of the single session on PBMT. Wavelength is also considered one of the most critical factors in all types of phototherapy, and the recommended wavelength should be 600 and 700 nm to treat superficial tissue. 25 31 32 Further studies are warranted to define the optimal wavelengths in the case of OLP healing. Regardless, there are not any recommendations or consensus reports by major associations/groups in the literature that can be considered a “Gold Standard” for PBMT procedures.

The meta-analysis of five studies that have assessed no difference in improvement in clinical score in patients treated with PBMT compared to the control regimens, including corticosteroids, emphasizes that PBMT is as effective as the standard regimens in managing OLP. In the study by Bhatt et al, the clinical severity score was reduced by 37.8% in the two months of treatment and by 37.2% during the follow-up period. 27 This result is consistent with another study by Cafaro et al, which showed a statistical significance in the difference in clinical scores after laser treatment. 33 However, depending on the lesions' characteristics, the number of laser sessions necessary for the tissues to heal was different. This could be due to the limited amount of RCT/NRCTs found. However, the results of this meta-analysis should not be taken as a firm conclusion. They should be interpreted cautiously because of the wide variety of study designs, laser parameters, and treatment outcomes in these investigations.

A considerable number of studies have demonstrated the role of PBMT in reducing the adverse effects of cytotoxic drugs on the oral mucosa by reducing inflammatory processes, reducing pain, preventing fibrosis, and improving wound healing, and tissue regeneration. 34 35 The safety of PBMT and the lack of any side effects make it a clear winner over other traditional treatments like corticosteroids. One of the studies showed that corticosteroids have detailed severe side effects such as burning and gastrointestinal distress. 22

The potential effects of PBMT on lowering the signs and symptoms of OLP can be linked to several processes at cellular and systemic levels. PBMT plays a vital role in the production of β-endorphins and encephalins and also reduces bradykinin and histamine levels, thereby contributing to the analgesic effect and pain relief. 36 The analgesic effect of PBMT has also been confirmed by its action on C-fibers, resulting in decreased C-fiber activity and reduced transmission of noxious stimuli. 37 The biological activity of PBMT in promoting enhanced proliferation, differentiation, and migration of fibroblasts and stimulation of epithelial cells, which are regarded as critical players in the healing process of the oral mucosa, could account for the decrease in clinical indications of OLP after treatment. 38 Moreover, PBMT also has an inherent mechanism to reduce inflammatory reactions by reducing the neutrophil infiltrates, leading to anti-inflammatory effects. 2 39 PBMT also makes collagen organization faster by stimulating collagen trihelix formation. 2 39 On the other hand, external factors, such as smoking, can affect the composition of inflammatory infiltrate in OLP, thus affecting immune surveillance. It has been shown that smoking could alter the inflammatory infiltrate by reducing the expression of macrophages (CD68 + ). 40 However, the exact impact of smoking on the action of PBMT has not been elucidated yet. PBMT also reduces the growth of several microbes, thus indirectly downregulating the associated inflammation in the oral microenvironment. 41 Thus, the complete mechanism of action of PBMT on oral tissue healing is yet to be elucidated.

Though our meta-analysis highlights the effectiveness of PBMT on OLP, the results of this study should be interpreted with caution. Primarily, heterogeneity was detected among the studies, which could be attributed to the different study designs involving different protocols, photosensitizers, and control interventions that prevented a standard protocol recommendation. Other than that, the sample size for subgroup analysis is limited, and the scarcity of studies that reviewed the effect of different types of treatment could increase the possibility of errors. The follow-up period varied between the studies, and this could affect the results. Despite these limitations, the study's findings give clinicians a thorough understanding of the effectiveness of PBMT in OLPs. However, more high-quality clinical trials are needed to increase the trustworthiness of the results. Suggested improvements for future research are the inclusion of well-designed RCTs with sufficient sample size and long-term follow-up, as well as the inclusion of standard laser parameters with suitable doses. Further efforts are also required to define the impact of PBMT on the malignant transformation of OLP.

## Conclusion

PBMT is as effective as other interventions in treating OLP, though not superior and without any adverse effects. Hence, it can be considered a promising alternative treatment for cases resistant to steroids or when steroids are contraindicated. Further studies are recommended to evaluate and standardize the optimal settings and follow-up period for the treatment of OLP.
