# Supplementary material for: Photobiomodulation Therapy in the Management of Oral Lichen Planus: A Systematic Review and Meta-Analysis
Source: Eur J Dent. 2024 May 14;18(4):976–86. doi: 10.1055/s-0044-1782213 (PMC11479744; doi:10.1055/s-0044-1782213)
Supplement: Supplementary file 1 — Supplementary Material [file 10-1055-s-0044-1782213-s2383050.pdf]

Supplementary Table S1 Search strategy in PubMed

Search no.	Searches	Hits
1	(“low level laser therapy” OR “laser phototherapy” OR “photobiomodulation therapy” OR “laser therapy” OR “laser treatment” OR “diode laser”)	61,732
2	(“oral potentially malignant disorder” OR “oral precancer” OR “oral premalignant” OR “ lichen planus” OR “leukoplakia”)	18,989
3	1 AND 2	318

Supplementary Table S2 Search strategy in Scopus

Search no.	Searches	Hits
1	TITLE-ABS-KEY (“low level laser therapy” OR “laser phototherapy” OR “photobiomodulation therapy” OR “laser therapy” OR “laser treatment” OR “diode laser”)	93,616
2	TITLE-ABS-KEY (“oral potentially malignant disorder” OR “oral precancer” OR “oral premalignant” OR “lichen planus” OR “leukoplakia”)	27,090
3	1 AND 2	435

Supplementary Table S3 Search strategy in Cochrane

Search no.	Searches	Hits
1	**(“low level laser therapy” OR “laser phototherapy” OR “photobiomodulation therapy” OR “laser therapy” OR “laser treatment” OR “diode laser”)**	114
2	(“oral potentially malignant disorder” OR “oral precancer” OR “oral premalignant” OR “lichen planus” OR “leukoplakia”)	6
3	1 AND 2	4

 © 2024. The Author(s).

Supplementary Table S4 Risk of bias for RCTs

	Random sequence generation (selection bias)	Allocation concealment (selection bias)	Blinding of participants and personnel (performance bias)	Blinding of outcome assessment (detection bias) (objective measures)	Incomplete outcome data addressed (attrition bias)	Selective reporting (reporting bias)	Other bias	Quality
Dillenburg et al (2014)[21]	Low	Low	High	High	Low	Unclear	Unclear	Poor
Kazancioglu and Erisen (2015)[24]	Low	Low	Unclear	Low	Low	Unclear	Unclear	Fair
Jajarm et al (2011)[25]	Low	Unclear	Low	Low	High	Low	Unclear	Fair
Bhatt et al (2022)[27]	Low	Low	Low	High	Low	Low	Unclear	Fair
Ferri et al (2021)[28]	Low	Low	Low	High	Hign	Unclear	Unclear	Poor
Mirza et al (2018)[22]	Low	Low	High	Low	Low	Unclear	Unclear	Poor
Jain et al (2021)[26]	Low	Low	Low	Low	Low	Unclear	Unclear	High

Abbreviations: RCTs, randomized controlled trials.

Supplementary Table S5 Risk of bias for observational studies

	Selection				Comparability	Exposure			Quality
	Is the case definition adequate?	Representativeness of the cases	Selection of controls	Definition of controls	Comparability of cases and controls on the basis of the design or analysis	Ascertainment of exposure	Same method of ascertainment for cases and controls	Nonresponse rate	
El Shenawy and Eldin (2015)[23]	*						*	*	Poor

 © 2024. The Author(s).

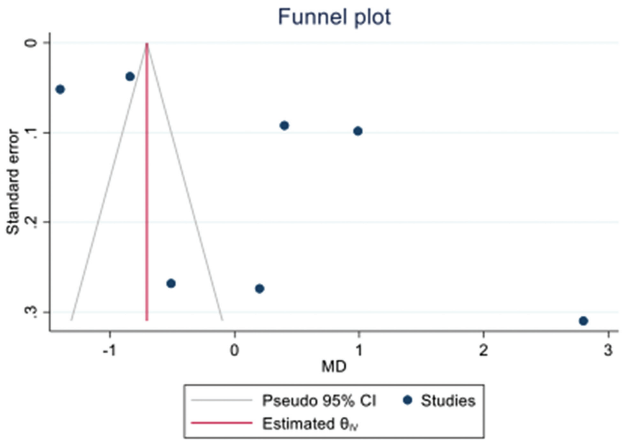

Supplementary Fig. S1 Funnel plot asymmetry test for the efficacy of photobiomodulation therapy on pain score (visual analog scale). CI, confidence interval; MD, mean difference.

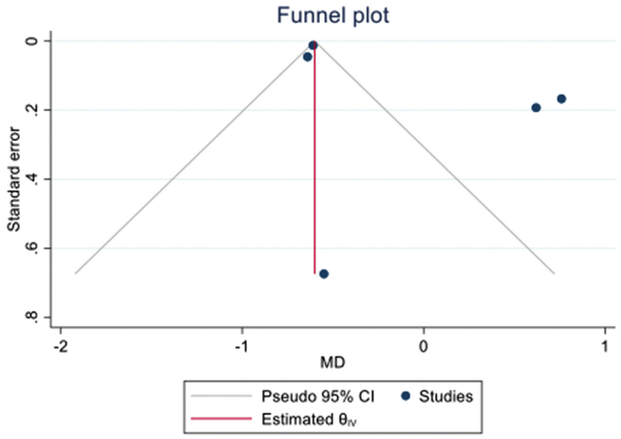

Supplementary Fig. S3 Funnel plot asymmetry test for the efficacy of photobiomodulation therapy on clinical severity. CI, confidence interval; MD, mean difference.

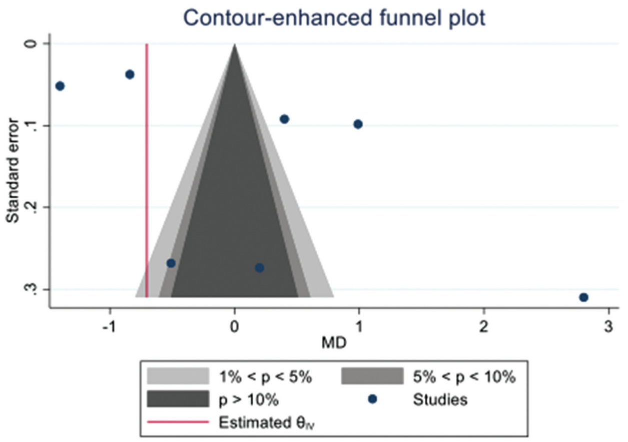

Supplementary Fig. S2 Contour-enhanced funnel plot for the efficacy of photobiomodulation therapy on pain score (visual analog scale). MD, mean difference.

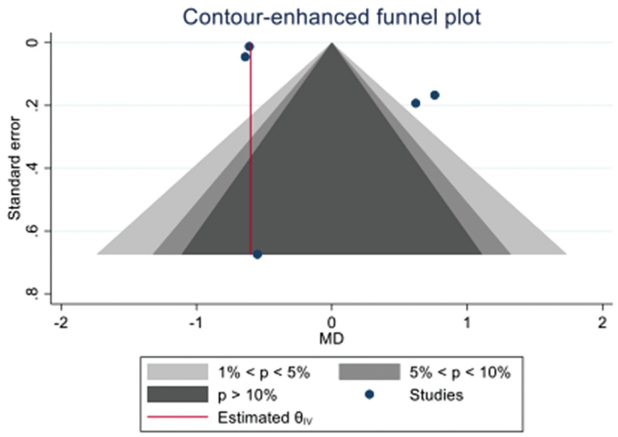

Supplementary Fig. S4 Contour-enhanced funnel plot for the efficacy of photobiomodulation therapy on clinical severity. MD, mean difference.

 © 2024. The Author(s).
